# Impact of the State of Emergency during the COVID-19 Pandemic in 2020 on Asthma Exacerbations among Children in Kobe City, Japan

**DOI:** 10.3390/ijerph182111407

**Published:** 2021-10-29

**Authors:** Hiroshi Yamaguchi, Kandai Nozu, Shinya Ishiko, Atsushi Kondo, Takeshi Ninchoji, China Nagano, Hiroki Takeda, Ai Unzaki, Kazuto Ishibashi, Ichiro Morioka, Hiroaki Nagase, Kazumoto Iijima, Akihito Ishida

**Affiliations:** 1 Department of Pediatrics, Kobe University Graduate School of Medicine, 7-5-2 Kusunoki-cho, Chuo-ku, Kobe 650-0017, Japan; nozu@med.kobe-u.ac.jp (K.N.); ishiko@med.kobe-u.ac.jp (S.I.); akondo@med.kobe-u.ac.jp (A.K.); nincho@med.kobe-u.ac.jp (T.N.); china@med.kobe-u.ac.jp (C.N.); hirotake@med.kobe-u.ac.jp (H.T.); nagase@med.kobe-u.ac.jp (H.N.); iijima@med.kobe-u.ac.jp (K.I.); 2 Kobe Children’s Primary Emergency Medical Center, 1-4-1 Wakihamakaigandori, Chuo-ku, Kobe 651-0073, Japan; ai.unzaki@gmail.com (A.U.); kztisbs@hotmail.com (K.I.); ishida@kobe-shyoniqq.jp (A.I.); 3 Department of Pediatrics and Child Health, Nihon University School of Medicine, 30-1, Oyaguchi, Kami-cho, Itabashi-ku 173-8610, Japan; morioka.ichiro@nihon-u.ac.jp

**Keywords:** Asian dust, air pollution, coronavirus disease, meteorology, pollen, respiratory infection, social isolation, typhoon

## Abstract

The coronavirus disease (COVID-19) pandemic altered environmental factors. We studied the impact of these changes on asthma exacerbation (AE) by comparing the AE-related environmental factors between COVID-19 (2020) and pre-COVID-19 (2011–2019) eras. Between 2011 and 2020, 278,465 children (<16 years old) visited our emergency department, and 7476 were diagnosed with AE. The number of patients showed spring and fall peaks in 2011–2019. Multivariate analyses showed significant positive relationships of the number of AE patients with the average temperature among all patients and 0–5-year-olds and with sulfur dioxide (SO_2_) levels in 2011–2019 among 0–5-year-olds. Although the spring peak in the number of patients was not observed in 2020 after declaration of a state of emergency, the fall peak was again observed after the state of emergency was lifted. No changes in average temperature were detected, but SO_2_ was significantly reduced following declaration of the state of emergency in 2020. Therefore, SO_2_ reduction might have contributed to the disappearance of the peak of AE. However, a fall peak was observed again in 2020, although SO_2_ levels continued to be low. These data suggest that person to person interaction seems to be associated with AE, presumably due to unknown viral infections.

## 1. Introduction

Asthma is characterized by bronchial hyperresponsiveness to a wide variety of stimuli, and its exacerbation is a major cause of morbidity [1]. Asthma exacerbation (AE), the temporary worsening of asthma symptoms, is multifactorial in etiology. Numerous studies have reported environmental factors for AE and such contributing factors include infections (e.g., a recent study in children reported that rhinovirus (RV) species were most frequently detected, followed by respiratory syncytial virus (RSV), influenza virus, human parainfluenza viruses, and human metapneumovirus) [2], meteorological factors (e.g., temperature, humidity, wind speed, and thunderstorms), and exposure to allergens (e.g., pollen, Asian dust) or pollutants (e.g., sulfur dioxide (SO_2_), nitrogen dioxide (NO_2_), particulate matters (PMs), carbon monoxide (CO), and ozone (O_3_)) [3,4,5,6,7,8,9,10,11,12,13,14,15,16]. Therefore, environmental factors are very important causes of AEs.

In 2020, the coronavirus disease (COVID-19) pandemic occurred worldwide [17]. In Japan, the first case of COVID-19 (adult) was reported on 16 January 2020. Elementary, junior high, and high schools were closed on 3 March 2020, and the Japanese government recommended that people stay home. As the number of infected people increased, the Japanese government issued an emergency declaration on 9 April 2020. The declaration was lifted on 25 May 2020, and schools resumed on 1 June 2020. Thereafter, avoiding social interaction and good hygiene, such as washing hands or wearing masks, have been recommended. This situation completely altered the exposure to environmental factors. Social distancing and the wide use of masks during the COVID-19 pandemic era have been reported to decrease the spread of severe acute respiratory syndrome coronavirus 2 (SARS-CoV-2), but also other respiratory viruses such as influenza or RSV [18]. Moreover, because of the limitations to everyday activities of individuals, industries, and companies during the COVID-19 era, the air quality improved in many cities, and reductions in SO_2_, NO, NO_2_, CO, and PM concentrations were observed [19,20]. These changes might have affected the incidence of AEs in our city, Kobe, Japan, and in fact, several studies describe a dramatic reduction in hospital visits or hospitalizations for AE following social isolation [21,22,23,24,25]. However, the durations of most previous studies were very short, covering a few to several weeks after lockdown. Therefore, it might be interesting to examine how environmental changes caused by the COVID-19 era before and after the emergency declaration affect the numbers of pediatric AE patients in one city over a longer study period such as the entirety of 2020.

The primary aim of the present study was to compare the numbers of pediatric patients with AE in 2020, the year of the COVID-19 pandemic, with those in previous years. The secondary aim was to examine the environmental factors, if they exist, contributing to the increase in the number of patients with AE by examining the association between environmental changes and the number of pediatric AE patients in 2020 compared to the era 2011–2019. To determine the environmental factors associated with AE in Kobe city, we first revealed the association between the number of pediatric patients with AE in previous years during 2011–2019 and exposure to multifaceted parameters, such as environmental factors (e.g., meteorological factors, air pollution, pollen, typhoon, and Asian dust) and viral infection prevalence (e.g., RSV and influenza) after controlling for confounding factors. As a sub-analysis, we analyzed infants and preschool children (0–5 years old) and school-aged children and adolescents (6–15 years old) separately because of the different clinical characteristics and prognoses of asthma in these populations [26]. Then, we compared the environmental factors, which were revealed to be associated with the increasing number of patients with AE, between the pre-COVID-19 era (2011–2019) and after (2020).

## 2. Materials and Methods

### 2.1. Study Design and Patients

This retrospective, clinical observational time-series study was conducted under the approval of the Ethics Committee of Kobe Children’s Primary Emergency Medical Center, with a waiver of informed consent owing to the retrospective nature of the study and accessibility to the public using our homepage (Approval No. 2019_5). The study protocol conformed to the ethical guidelines of the 1975 Declaration of Helsinki.

This study was performed in Kobe city, which is located in the southern part of the main island of Japan. The characteristics of Kobe Children’s Primary Emergency Medical Center were previously described [27]. Briefly, our center provides medical services for pediatric patients during holidays and outside of regular working hours (7:30 p.m. to 7:00 a.m.) and is the largest nighttime emergency medical center in the Hyogo Prefecture, Japan. We reviewed the clinical database of consecutive patients < 16 years old at the ED of Kobe Children’s Primary Emergency Medical Center between 1 January 2011, and 31 December 2020. Data included information on patients’ sex, age, diagnosis, and date of visit. In total, 278,465 children visited the center during the study period, and a total of 7476 children who visited the center during the night shift (7:30 p.m. to 7:00 a.m.) were diagnosed with AE (ICD-10 codes: status asthmaticus (J46), bronchial asthma attack (J46), infectious bronchial asthma (J451), bronchial asthma (J459), and childhood asthma (J450)) by pediatricians in the center and included in the present study (Table 1). To estimate the prevalence of viral infections, we also counted the number of patients diagnosed with RSV and influenza infections in our center. Viral data were focused on RSV and influenza because these two viruses are commonly diagnosed by rapid antigen tests in Japan. The daily number of cases of each virus was collected.

### 2.2. Meteorological Data

Meteorological data of Kobe city from 2011 to 2019 in the present study were the same data retrieved for our previous study from the Japan Meteorology Agency (https://www.jma.go.jp/jma/indexe.html; accessed on 14 August 2020) [27]. This agency publishes on its homepage daily meteorological data which can be imported into Excel. In this study, the data for 2020 were newly retrieved from the same agency. Daily atmospheric pressure (hPa), precipitation (mm), temperature (°C), humidity (%), wind speed (m/s), and hours of sunlight (h) were obtained. The days of typhoon landing were also obtained from this agency (https://www.data.jma.go.jp/fcd/yoho/typhoon/statistics/index.html; accessed on 14 August 2020). Kobe City faces the Rokko Mountains to the north and Osaka Bay to the south, is warm and receives light rain throughout the year, and belongs to the Humid subtropical climate. Among the environmental pollutants included in our research, SO_2_, NO_2_, OX, SPM, and PM_2.5_ have environmental standards in Japan (Environmental reference value: SO_2_, the daily average of 1-h values is ≤0.04 ppm, and the 1-h values are ≤0.1 ppm; NO_2_, the daily average of hourly values should be within or below the zone from 0.04ppm to 0.06ppm; OX, the 1-h value is ≤0.06 ppm; SPM, the daily average of 1-h values is ≤0.10 mg/m^3^, and the 1-h values are ≤0.20 mg/m^3^; PM_2.5_, the annual average value is ≤15 μg/m^3^, and the daily average value is ≤35 μg/m^3^) (https://www.nies.go.jp/igreen/explain/air/kan.html; accessed on 14 August 2020: National Institute for Environmental Studies). SO_2_ is generated when coal, petroleum, etc. are burned. It is mainly discharged from factories, such as steelworks, and causes respiratory disorders, such as asthma. NO is mainly emitted into the atmosphere from factories and automobiles. NO_2_ is primarily generated from the combustion process of boilers or automobiles and causes respiratory disorders. NOX is mainly emitted from factories, thermal power plants, and automobiles and causes respiratory disorders. OX is generally emitted into the atmosphere from factories and automobiles and causes photochemical smog. It stimulates the eyes and the respiratory tract. THC and methane are mainly emitted by burning fossil fuels and agricultural activities and contribute to greenhouse gases. NMHC is mainly emitted from automobiles and oil refineries. SPM is particulate matter suspended in the atmosphere with a particle size of 10 μm or less and is emitted from factories, automobiles, volcanoes, etc. PM_2.5_ is in the atmosphere with a particle size of 2.5 μm or less and is mainly emitted from factories, automobiles, ships, aircraft, and volcanoes. It mainly affects the respiratory tract.

### 2.3. Air Pollutants Data

Daily data on air pollutants from 2011 to 2019 in the present study were the same data used in the previous study [27]. Kobe city publishes daily air pollutant data on its homepage (Hyogo Nanbu Taiki Sokutei Kyoku, http://kobe-taikikanshi.jp/kankyo/; accessed on 14 August 2020), which can be imported into Excel. In this study, the data for 2020 were newly retrieved from the same station, which is one of the main stations in Kobe city (6.05 km from our medical center). The data of SO_2_, NO, NO_2_, NOX, OX, CH_4_, NMHC, THC, SPM (PM ≤ 10 μm), and PM_2.5_ were obtained. PM_2.5_ measurements began on July 30, 2012. Therefore, PM_2.5_ data were included thereafter. The days with Asian dust observed in Kobe city were retrieved from the Kobe city homepage (https://www.city.kobe.lg.jp/a66324/kurashi/recycle/kankyohozen/air/kousa.html; accessed on 14 August 2020).

### 2.4. Pollen Data

Daily pollen data were collected for the same period from “Hanako-san”, the Japanese Ministry of the Environment pollen observation system (http://kafun.taiki.go.jp/; accessed on 14 August 2020). Only data collected from February to May were available because of the increased number of patients with pollen allergies during this period in Japan [28].

### 2.5. Statistics

Results are expressed as the number (%) or mean (standard deviation (SD)). Student’s *t*-test, a Chi-squared test or one-way analysis of variance was used, as appropriate, for statistical analysis of the results. Correlations were analyzed using Pearson’s correlation coefficient. These analyses were performed using GraphPad Prism 5.0 (GraphPad Software, San Diego, CA, USA).

Using EZR (ver. 1.50), multivariate analyses of Poisson regression estimates were conducted to investigate the effects of multifaceted meteorological factors, air pollutants, pollen, and viral infections on the number of AE patients. To reduce the Akaike’s information criterion (AIC) value, we excluded strongly correlated (Pearson’s r > 0.8) values from the model. In general, the smaller the AIC value, the better the fit for the model of multivariate analyses of Poisson regression estimates. EZR is a graphical user interface for R (The R Foundation for Statistical Computing) based on the R commander; it can be downloaded for free (http://www.jichi.ac.jp/saitama-sct/SaitamaHP.files/statmed.html; accessed on 14 August 2020) and is used widely for medical statistics [29]. A two-tailed *p*-value < 0.05 was considered statistically significant.

## 3. Results 

### 3.1. Patient Demographics

The number of patient visits for AE and the total number of patients each year are shown in Table 1. During the study period, 7476 children < 16 years old were diagnosed with AE. Between 1 January 2011, and 31 December 2020, the mean (standard deviation (SD)) age was 5.7 (3.3) years, and 64.0% were boys. A total of 4495 (60.1%) AE events were reported in infants and preschool children (0–5 years old), and 2981 (39.9%) AE events were reported in school-aged children and adolescents (6–15 years old). In 2020, the daily number of patient visits for AE significantly declined compared to previous years (average (SD): all patients, 1.8 (1.7) vs. 0.9 (1.3), *p* < 0.0001; <6 years, 1.1 (1.2) vs. 0.6 (0.9), *p* < 0.001; ≥6 years, 0.7 (1.0) vs. 0.4 (0.7), *p* < 0.0001; Student’s *t*-test). However, the average proportion of patients with AE from 2011 to 2019 did not differ from that of 2020 (2.7 (2011–2019) vs. 2.6 (2020), *p* = 0.38; Chi-squared test).

### 3.2. Monthly Changes in the Number of Patients with AE in Kobe, Japan

**Figure 1:**
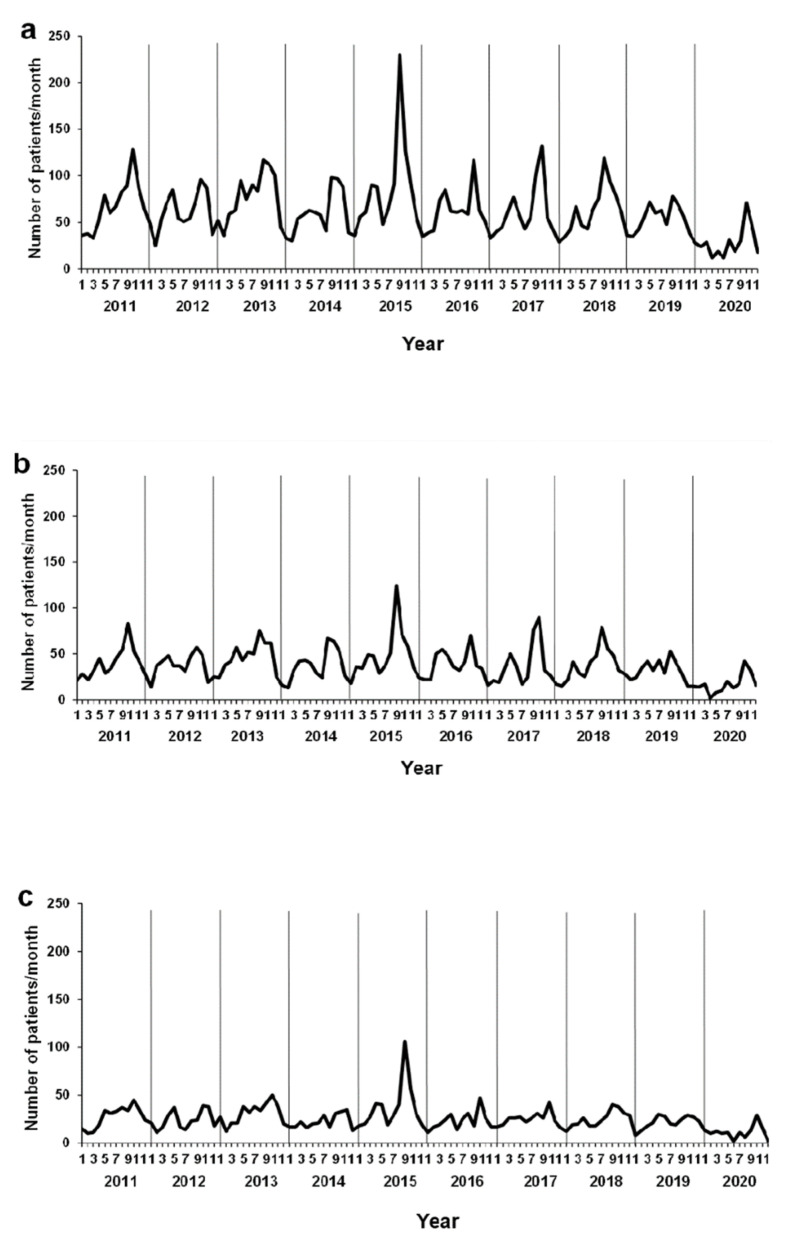
Observed numbers of emergency department visits for asthma exacerbation per month as a function of time. Data are presented by year: (**a**) total population, (**b**) 0–5 years old, and (**c**) 6–15 years old.

The monthly changes in the number of patients with AE are shown in Figure 1. The number of patients with AE from 2011 to 2019 showed distinct seasonal changes with dominant peaks during spring (April and May) and fall (September and October) (Figure 1a). The fall peak was generally higher than the spring peak. In 2020, the dominant peak during spring (April and May) observed in previous years was not found. During this period, schools were closed, and the Japanese government urged people to stay home. The fall peak was observed similar to that in a previous year (2019). During this period, schools were open. Sub-analysis stratifying patients by age (0–5- and 6–15-year-old groups) revealed that these two seasonal peaks were more prominent in the 0–5-year-old group than in the 6–15-year-old group during 2011–2019 (Figure 1b,c). As there was an enterovirus D68 outbreak during the fall peak in 2015, the highest peak occurred during fall in 2015 [30]. The spring peak of patients with AE was observed in neither the 0–5-year-old nor the 6–15-year-old group in 2020. However, the fall peak was similar to that in a previous year (2019) in both groups. 

### 3.3. Background of Environmental Factors and Viral Infections

To elucidate the environmental factors and viral infections for AE among children in Kobe city, these data were analyzed for the period 2011–2019. Seasonal mean values of meteorological factors and air pollutants are shown in Table 2. The mean values of each factor were calculated for each season. Between 1 January 2011, and 31 December 2019, the mean atmospheric pressure was high in winter and low in summer. In contrast, the mean values of precipitation and humidity were high in summer and low in winter. The average temperature ranged from 7.0 °C (winter) to 26.6 °C (summer). Hours of sunlight were the longest in spring and the shortest in winter. The average wind speed did not show any noticeable fluctuation. 

Air pollutant variables in Kobe, Japan, during the study period are shown in Table 2. Between 1 January 2011, and 31 December 2019, the seasonal air pollutant variables that were the highest during spring, summer, and winter were NO_2_, oxidant (OX), and PM_2.5_; SO_2_ and suspended particulate matter (SPM); and nitric oxide (NO), nitrogen oxide (NOX), methane (CH_4_), non-methane hydrocarbons (NMHC), and total hydrocarbons (THC), respectively. Seasonal air pollutant variables that were lowest during summer, fall, and winter were NO_2_, NOX, CH_4_, NMHC, and THC; SO_2_, NO, and PM_2.5_; and OX and SPM, respectively.

The pollen levels observed in Kobe are shown in Table 3. Between 1 January 2011, and 31 December 2019, the mean pollen counts were high in 2019 and low in 2012. The total average pollen count was highest in April.

**Figure 2:**
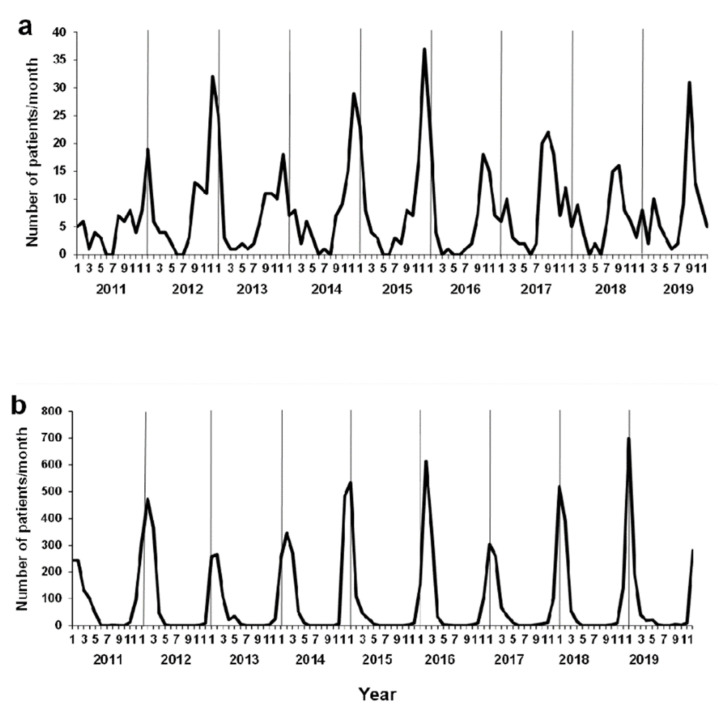
Seasonal changes of viral respiratory tract infections due to respiratory syncytial virus (RSV) and influenza. Seasonal changes of (**a**) RSV and (**b**) influenza infections diagnosed in our emergency department.

Seasonal changes for viral respiratory tract infections in our emergency department (ED) are shown in Figure 2. The number of patients with RSV and influenza infections showed distinct maxima and minima (Figure 2a,b). Although the peak of RSV infection was observed during winter for 2011–2015, the peak shifted to fall thereafter; this trend was also seen nationwide in Japan (https://www.niid.go.jp/niid/images/iasr/rapid/topics/rsv/150918/rsv1_191215.gif; accessed on 14 August 2020).

### 3.4. Association between Asthma ED Visits and Environmental Factors

To reveal the association of asthma ED visits with environmental factors and infections, we analyzed these data between 1 January 2011, and 31 December 2019.

**Figure 3:**
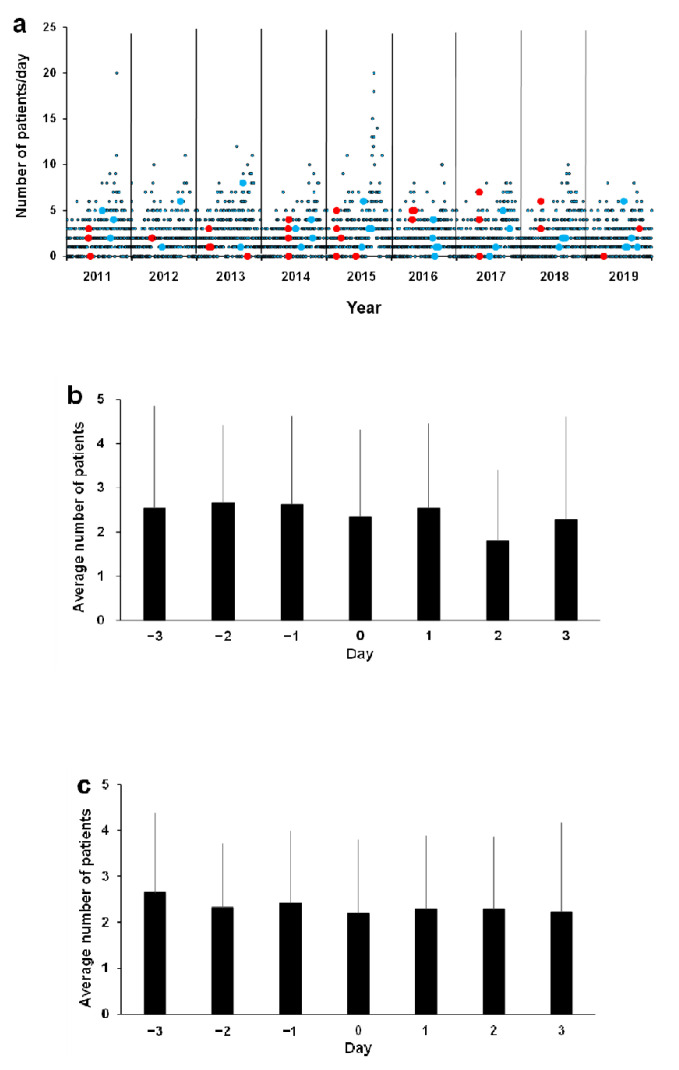
The days with typhoon landings and Asian dust observations are plotted as observed numbers of emergency department visits for asthma exacerbation (AE) per day as a function of time (**a**). Red and green dots indicate the numbers of AE patients when a typhoon landed or Asian dust was observed, respectively. The numbers of AE patients during the three days before and after the event are shown for typhoon landings (**b**) and Asian dust (**c**). In both cases, there are no changes in the number of AE patients during the three days before and after the event. Data are presented as daily mean patients plus standard deviation.

The days of typhoon landing and Asian dust observation were plotted along with observed numbers of ED visits for AE per day as a function of time (Figure 3a). In both cases, there was no change in the number of patients with AE during the three days before and after the event (Figure 3b (typhoon) and Figure 3c (Asian dust)). There were no significant differences (typhoon *p* = 0.59, Asian dust *p* = 0.95; one-way analysis of variance). Pearson correlation coefficients between environmental variables are shown in Table 4. We found significant correlations (Pearson’s r > 0.8) of the average temperature with the highest and lowest temperatures, the highest with the lowest temperature, NO with NO_2_ and NOX, NOX with NMHC, NMHC with THC, and SPM with PM_2.5_. The multivariate analysis of Poisson regression estimates measuring these associations is shown in Table 5. A significant positive relationship of the number of AE patients per day with the average temperature was observed in all age groups (relative risk (RR), 1.03; 95% confidential interval (CI), 1.01–1.05) and in infants and preschool children (0–5 years old: RR, 1.03; 95% CI, 1.01–1.06), as well as with SO_2_ in infants and preschool children (0–5 years old: RR, 1.08; 95% CI, 1.005–1.16).

**Figure 4:**
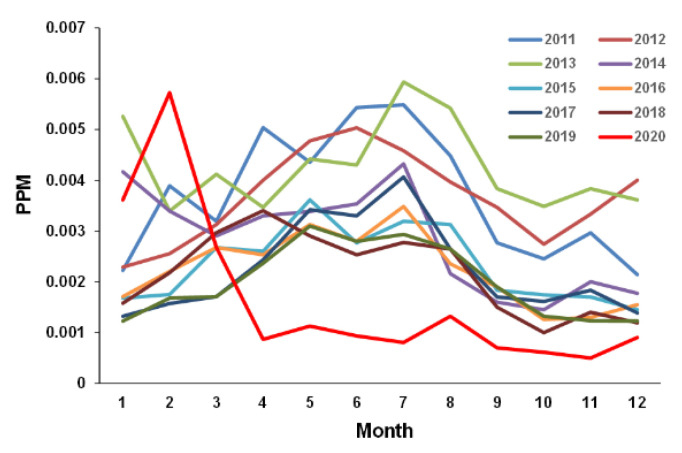
Seasonal dynamics of sulfur dioxide (SO_2_) per month. Monthly mean of SO_2_ in 2011–2020.

In 2020, the dominant peak of the number of AE patients during spring observed in previous years was not found. To compare the situation between 2020 and previous years, statistically significant environmental factors, temperature and SO_2_, were compared across years. The average temperature in 2020 was not significantly different compared to that in previous years (average (SD) 17.2 (8.8) °C in 2011–2019 vs. 17.6 (7.7) °C in 2020, *p* = 0.08, Student’s *t*-test). However, SO_2_ in 2020 was significantly lower than the value in the previous year (0.0020 ppm in 2019 vs. 0.0016 ppm in 2020, *p* = 0.034, Student’s *t*-test). Notably, although there was a clear year-to-year variation in SO_2_ from 2011 to 2019 (Figure 4), which was particularly high in May–August (i.e., about twice as high as in winter), SO_2_ decreased after a nationwide emergency declaration was issued on 9 April 2020. Such air pollution declines during COVID-19 lockdowns have been observed worldwide [31,32,33].

## 4. Discussion

We studied the impact of the COVID-19 pandemic on the number of AE patients by comparing the environmental factors between the COVID-19 (2020) and pre-COVID-19 (2011–2019) eras. Most studies have shown a similar reduction in the number of AE patients after lockdown, but past studies were only short-term, whereas our study has a relatively long observation period of one year [34,35]. To the best of our knowledge, only one recent study described pediatric AE case numbers over the year 2020 and showed the absence of the typical fall seasonal spike in Washington, DC, USA [36]. Therefore, we consider our research to be worth reporting. In the present study, we found that the number of pediatric patients with AE in 2020 significantly decreased compared to that in previous years. Although its spring peak observed in previous years was not found in 2020, the fall peak observed in 2020 was similar to those in previous years. Second, we found significant positive relationships between the average temperature and the number of patients with AE in all age groups and infants and preschool children (0–5 years old), as well as SO_2_ in infants and preschool children (0–5 years old) during the pre-COVID-19 era (2011–2019). The average temperature in 2020 was similar to that in previous years, whereas SO_2_ was significantly lower in 2020. These results suggest that the reduction in SO_2_ was related to the reduction in the number of patients with AE during spring 2020. However, in addition to the reduction in the number of older children with AE (6–15 years old) during spring (SO_2_ was found not to be related to the number of patients for those ages), and the observation of a fall peak in 2020 as usual even though SO_2_ remained low, other factors, such as social isolation and interaction, may have contributed to the disappearance of the peak incidence during spring and reappearance during fall.

In clinical practice, patients with asthma sometimes describe exacerbation of asthma due to changes in climate. Previous studies reported meteorological factors (e.g., temperature, humidity, wind speed, thunderstorm) for AE [6,9,10,11,14,15,37]. However, studies about the relationship between AE and changes in climate-related parameters have been inconsistent. As for pediatric AE patients, meteorological factors, such as temperature, diurnal temperature range, relative humidity, and wind speed, were significantly associated with ED visits for childhood AE in China [14,15]. Diurnal temperature range and temperature fluctuations were reported as risk factors in Korea, whereas a study in Japan found no correlation with meteorological parameters [6,10]. In the present study, an increased temperature was one of the risk factors for AE in children. Mohr et al. reported that a 0.10 μg/m^3^ increase in elemental carbon resulted in a 9.45% increase in asthma ED visits during the summer, and this risk increased with increasing temperature [11]. Lee et al. reported that the diurnal temperature range had significant effects on pediatric patients [6]. Ueda et al. reported that within-day temperature changes rather than the ambient temperature were associated with AE [9]. However, Abe et al. reported no significant associations between temperature and AE among children in Tokyo, Japan [10]. In the present study, a positive relationship was found between patients with AE and the average temperature. We hypothesize that the reason for such inconsistent results is that children in developed countries live in environments where the temperature is well controlled due to modern architecture and the widespread use of air conditioners. In addition, Deng et al. recently reported that in a mouse model, both high and low temperatures can aggravate asthma-induced airway inflammation [38]. Therefore, meteorological factors seem to have complicated effects on AE. Previous studies have described thunderstorm asthma, an asthma outbreak characterized by a sudden increase in hospital visits due to AEs concomitant with thunderstorms [39]. The mechanism of thunderstorm asthma is still unknown. Some factors have been considered for thunderstorm asthma, such as high concentrations of aeroallergens, strong winds, or heavy rains [40,41]. However, some studies reported no association between thunderstorms and outbreaks of AE [42]. In the present study, we also did not find any apparent associations between typhoons and AE.

Previous studies also showed a positive relationship between the number of patients with AE and air pollutants [43,44,45,46,47]. In the present study, SO_2_ was positively associated with the number of patients among infants and preschool children (0–5 years old) with AE. SO_2_ is an environmental toxicant promoting airway responses in a concentration-dependent manner, possibly by local oxidative stress [48]. Kuo et al. reported that the level of SO_2_ was most strongly associated with daily asthma hospitalizations of children among all the pollutants studied [49]. This finding is consistent with our study results. The environmental reference value of SO_2_ in Japan is the daily average of 1-h values ≤ 0.04 ppm, and the mean value of SO_2_ in the present study was 0.0028 ppm (2011–2019). Compared to previous studies (0.00491 ppm in Athene, Greece [6]; 0.018 ppm in Atlanta, USA [50]), the SO_2_ level in Kobe city was low and tended to decline even before the COVID-19 pandemic (Figure 4). These data suggest that SO_2_ affects the respiratory tract of younger children, such as preschool children (0–5 years old).

In addition to SO_2_, the air pollutants that were frequently associated with AE were NO_2_, O_3_, CO, and PM [6,22,43,51,52,53]. The differences in the results of epidemiologic studies regarding the effects of air pollution on AE may be due to the environmental diversity in each region. In addition, Lovinsky-Desir et al. emphasized the impact of social stressors, such as poverty, public assistance, or crowded housing, on AE [5]. Social factors, such as parental stress or violence, have been reported to be associated with AE [54,55]; therefore, it is considered that many factors are intertwined. 

Regarding other environmental factors, some studies previously reported an association between AE and Asian dust. Asian dust is mineral dust transported by wind from the Gobi and Taklimakan deserts of China and Mongolia to the Pacific. These studies showed no significant association between AE and Asian dust [9,56,57], which is consistent with our findings. In addition, there are several reports on the relationship between pollen and asthma in children [6,58], showing that pollen was also an important trigger of AE in children. In Japan, *Cryptomeria japonica* (sugi; Japanese cedar) and *Chamaecyparis obtusa* (hinoki; Japanese cypress) are commonly grown, and these trees produce allergenic pollen. The pollen dispersal season occurs mainly from February to May [59]. Therefore, data were only obtained during this period in previous studies. In the present study, an increased number of patients with AE was observed in April and May, during pollen dispersal. However, Poisson regression estimates revealed no relationship in the present study. Our study found no relationship between the number of patients with AE and the prevalence of RSV or influenza infections. Numerous studies have reported on the association between AE and infections, especially viral infections, which are most common and considered as triggers for up to 90% of AEs [3,58,60,61]. RSV has been reported as a frequent cause of AE [3,62,63]. In Japan, a seasonal rise in RSV infection usually starts in September (autumn season). In the present study, it was not possible to directly prove that RSV infection contributed to AE. The association between influenza and AE is controversial [58,64,65]. Our study suggests that the number of AEs decreases during the winter when influenza is prevalent; therefore, influenza is less likely involved.

The reasons for the decrease in the number of patients during spring in 2020 were probably as follows: (1) avoidance of visiting the ED because of fear of contracting a COVID-19 infection in the ED, and (2) decreased exposure to outdoor environmental stressors or infections due to social distancing [66]. As for the first reason, AE is a potentially fatal disease, and it is reasonable to assume that families would have visited the hospital had their children developed severe cough or dyspnea because patients with mild AE did not visit the ED and deteriorated. However, our center did not experience an increased number of patients with severe AE after the state of emergency. The same situation was observed in London, UK [66]. Therefore, this reason may be less likely. As for the second reason, the reduction in SO_2_ might partially contribute to the decreased number of AE patients in 0–5-year-olds because we found that SO_2_ is one of the risk factors for AE in 0–5-year-olds. However, the number of AE patients older than 6 years of age also decreased during this period. Therefore, the cause of the result is still unknown. We also found that the average proportion of patients with AE from 2011 to 2019 did not differ from that of 2020. If SO_2_ solely contributes to the reduction in AE, the proportion of patients with AE may decrease. As the chief complaint of most patients in our primary center was fever, it may be reasonable to consider that by reducing the number of infectious diseases by social isolation, the total number of patients and the number of patients with AE were reduced. Our study findings also suggest that although there are several AE-inducing indoor allergens, such as rodents, cockroaches, domestic animals, and mold [67], the effect of outdoor stressors is probably stronger than that of indoor stressors because many children stayed at home during the state of emergency, but the number of asthma patients did not increase. Another interesting finding is that the number of patients with AE showed the usual increase in fall. COVID-19 itself has been reported to be related to AE [68,69]. However, in 2020, only a few COVID-19 cases in patients under 20 years of age were reported in Kobe (<10 years old, n = 3 (Apr), n = 7 (Jul), n = 20 (Aug), n = 7 (Sep), n = 6 (Oct), n = 12 (Nov), and n = 34 (Dec); ≥10 to <20 years old, n = 1 (Mar), n = 6 (Apr), n = 7 (Jul), n = 26 (Aug), n = 28 (Sep), n = 14 (Oct), n = 61 (Nov), and n = 71 (Dec)) (https://www.city.kobe.lg.jp/a73576/kenko/health/infection/protection/covid_19.html; accessed on 14 August 2020). Therefore, COVID-19 itself may have a minimal effect on AE. The most likely reason for increased numbers of pediatric AE patients during fall may be due to RV infections. Johnston et al. reported that hospital admission rates for AE in school-aged children correlate with the seasonal increase in RV infections from September to December and again in the spring [70]. This seasonal RV prevalence coincides with the number of AE patients during fall in the present study. As RVs cause usually mild symptoms of the common cold, patients in Japan are not generally tested for RV. Therefore, our retrospective study could not analyze the relationship between RV infection and the number of patients with AE. Takashita et al. reported RV infections in children during the COVID-19 pandemic in Japan [71]. They clearly showed increased bimodal RV infections in spring and autumn until 2019, but a reduction in RV infections during spring 2020 with a dramatic increase during fall 2020 [71]. Fall peaks may also have been caused by other viral infections that were not included in their investigation. In 2015, a significantly increased peak was observed during fall and associated with enterovirus D68 infections [24]. Therefore, viral infections may play an important role in the fall peak observed in our study. However, we could not elucidate the reasons for the peak in pediatric patients with AE during the fall season of 2020. Further research is needed.

The present study has several limitations. First, the diagnosis of AE was at the discretion of the doctors who consulted with the patient. Due to the retrospective nature of this study, there was a lack of strict diagnostic criteria for asthma. Second, our database did not include other factors, such as medical history or medication. Therefore, these data were unknown. Third, regarding air pollutants, the data of only one center (the most stable measurement in the city) was chosen, and they may differ from the actual environment of patients. Fourth, there might have been selection bias regarding the patients with AE. However, our center is the only one that provides primary medical care at night and during holidays for the population of more than 1.5 million people in Kobe city. We included all cases visiting our center between 1 January 2011, and 31 December 2020 (10 years). As a result, this study included the large number of AE patients so far (n = 7476); thus, population heterogeneities may have been minimized by this large number of patients. Finally, our study did not consider other viruses, such as RV, parvovirus, paramyxovirus, coronavirus, parainfluenza, adenovirus, and metapneumovirus, which have also been reported to have an impact on AE [3,65,72]. In addition, in 2020, it was not common to test for COVID-19 in Japan. In particular, as AE patients do not always have fever, our center did not test AE patients for COVID-19. Therefore, it is unclear how many AE patients were exacerbated by COVID-19. However, as described in our manuscript, the number of patients with COVID-19 in Kobe in 2020 was very small, suggesting that very few patients had AE induced by COVID-19. Despite these limitations, this study has a relatively large sample size and comprehensively investigates asthma triggers.

## 5. Conclusions

The present study examined the effect of the COVID-19 pandemic on the number of pediatric patients with AE. After the state of emergency was declared during spring in 2020, the number of pediatric patients with AE dramatically decreased. However, after the state of emergency was lifted, the fall peak of AE was observed as usual. Although the reduction in SO_2_ was partially related to the reduction in the number of patients with AE in 2020, social isolation and interaction may have strongly contributed to the decrease and increase in the number of patients with AE. Social isolation reduced exposure to environmental factors and infections, thereby decreasing the number of patients with AE. The resumption of social interaction led to re-exposure to environmental factors and infections, thus increasing the number of patients with AE. 

## Figures and Tables

**Table 1:** The number of patient visits for asthma exacerbation (AE) and the total number of patients each year (2011–2020).

Year	Patient Visits for AE			All Patients
	Total	0–5 Years Old	6–15 Years Old	Total
2011–2020	7476	4495 (60.1%)	2981 (39.9%)	278,465
2011	817	489	328	30,888
2012	734	447	287	30,885
2013	929	555	374	29,340
2014	720	449	271	30,580
2015	1034	590	444	29,426
2016	750	471	279	30,106
2017	744	443	301	26,884
2018	755	453	302	27,751
2019	653	392	261	29,331
2020	340	206	134	13,274

**Table 2:** Environmental variables in Kobe, Japan (2011–2019).

Variable	Overall	Spring (Mar–May)	Summer (Jun–Aug)	Fall (Sep–Nov)	Winter (Dec–Feb)
**Meteorological data**					
Atmospheric pressure (hPa)	1011.5	1011.4	1005.2	1012.8	1016.6
Precipitation (mm)	3.9	3.5	5.7	4.5	1.7
Average temperature (°C)	17.2	15.1	26.6	19.8	7.0
Highest temperature (°C)	20.7	19.0	30.0	23.2	10.3
Lowest temperature (°C)	14.1	11.5	24.0	16.9	4.0
Humidity (%)	64.9	61.3	72.2	65.1	61.1
Average wind speed (m/s)	3.7	3.6	3.6	3.8	3.8
Hours of sunlight (h)	5.8	6.6	6.4	5.3	5.0
Highest temperature − lowest temperature (°C)	6.5	7.5	6.0	6.3	6.3
Average temperature difference (day − previous day, °C)	6.5	7.3	5.9	6.5	6.3
**Air pollutants**					
SO_2_ (ppm)	0.0028	0.0032	0.0037	0.0021	0.0023
NO (ppm)	0.0039	0.0037	0.0038	0.0031	0.0052
NO_2_ (ppm)	0.016	0.018	0.013	0.015	0.017
NOX (ppm)	0.020	0.022	0.017	0.018	0.022
OX (ppm)	0.029	0.037	0.027	0.027	0.023
CH_4_ (ppmC)	1.92	1.93	1.86	1.92	1.96
NMHC (ppmC)	0.11	0.12	0.10	0.11	0.12
THC (ppmC)	2.02	2.04	1.96	2.02	2.07
SPM (mg/m^3^)	20.3	22.5	26.5	16.5	15.6
PM_2.5_ (µg/m^3^)	13.7	16.4	14.1	11.2	13.5

Data are represented as means. Data are missing on the following dates due to maintenance of measuring instruments or suspension of measurement due to reconstruction of the station building: SO_2_ 4/8–11, 12/23–25/2011, 2/25–26/2012, 3/9–3/14/2013, 1/13–19/2014; NO 5/5–28, 6/3–9/11/2013, 1/20–27, 2/1–2, 2/6–9/2014, 12/23/2015, 5/30–6/5, 8/20–9/15, 9/19–29, 11/15, 12/2–12/6, 12/15–20/2016, 9/5–9/11/2017, 9/12–16, 10/1, 12/6–9, 12/17–18, 12/24–30/2018, 2/26, 6/4/2019; NO_2_ 5/5–28, 6/4–9/11/2013, 1/20–27, 2/1–2, 2/6–9/2014, 12/23/2015, 5/30–6/5, 8/20–9/15, 9/19–29, 11/15, 12/2–12/6, 12/15–20/2016, 9/5–9/11/2017, 9/12–16, 10/1, 12/6–9, 12/17–18, 12/24–30/2018, 2/26, 6/4/2019; NOX 5/5–28, 6/3–9/11/2013, 1/20–27, 2/1–2, 2/6–9/2014, 12/23/2015, 5/30–6/5, 8/20–9/15, 9/19–29, 11/15, 12/2–12/6, 12/15–20/2016, 9/5–9/11/2017, 9/12–16, 10/1, 12/6–9, 17–18, 24–30/2018, 2/26, 6/4/2019; OX 8/20, 11/25–27/2019; CH_4_ 9/1–2, 11/28/2013, 2/13–16, 7/18–19/2016, 10/4–8/2019; NMHC 9/1–2, 11/28/2013, 2/13–16, 7/18–19/2016, 10/4–8/2019; THC 9/1–2, 11/28/2013, 2/13–16, 7/18–19/2016, 10/4–8/2019; SPM 10/11–14/2013. Abbreviations: ppmC, concentration unit of parts per million carbon; hPa, hectopascal; SO_2_, sulfur dioxide; NO, nitric oxide; NO_2_, nitrogen dioxide; NOX, nitrogen oxides; CH_4_, methane; NMHC, non-methane hydrocarbons; THC, total hydrocarbons; SPM, suspended particulate matter; PM_2.5_, particulate matter (2.5 μm).

**Table 3:** Pollen levels in Kobe, Japan (Feb–May 2011–2019).

Annual Amount (Average)	
Year	(/mm^3^/day)
2011	19.6
2012	11.7
2013	12.6
2014	20.3
2015	14.7
2016	20.1
2017	13.4
2018	15.0
2019	21.1
**Average Monthly Value (2011–2019)**	
**Month**	**(/mm^3^** **/day)**
Feb	6.7
Mar	20.5
Apr	23.2
May	14.8

Data are missing on the following dates due to maintenance of measuring instruments or suspension of measurement due to reconstruction of the station building: 5/2–4, 5/13/2011, 4/24–25/2012, 3/9–10, 3/19–3/20/2013, 2/20, 2/23–25, 3/22, 4/25–26, 5/9, 5/29–31/2014, 2/20, 2/23–25, 3/22, 4/25–26, 5/9/2015, 2/20, 4/24–25, 5/8/2016, 4/16–17/2018.

**Table 4:** Pearson’s correlation coefficients between environmental variables.

	Meteorological Data									
	Atmospheric Pressure	Precipitation	Average Temperature	Highest Temperature	Lowest Temperature	Humidity	Wind Speed	Hours of Sunlight		Temperature Difference (Day − Previous Day)
**Meteorological data**										
Atmospheric pressure	1.0									
Precipitation	−0.3167 ****	1.0								
Average temperature	−0.6119 ****	0.1146 ****	1.0							
Highest temperature	−0.5896 ****	0.08268 ****	0.9919 ****	1.0						
Lowest temperature	−0.6232 ****	0.1352 ****	0.992 ****	0.9734 ****	1.0					
Humidity	−0.4641 ****	0.4461 ****	0.4094 ****	0.3462 ****	0.4588 ****	1.0				
Wind speed	−0.2359 ****	0.2744 ****	−0.0367 *	−0.04834 **	−0.01835	0.02506	1.0			
Hours of sunlight	0.1584 ****	−0.3538 ****	0.1375 ****	0.215 ****	0.07075 ****	−0.5122 ****	−0.1913 ****	1.0		
Temperature difference	0.1931 ****	−0.2355 ****	−0.07914 ****	0.03676 *	−0.1933 ****	−0.5182 ****	−0.1269 ****	0.6116 ****		1.0
(day − previous day)										
**Air pollutants**										
SO_2_	−0.1529 ****	−0.07922 ****	0.2868 ****	0.2991 ****	0.2656 ****	0.1642 ****	−0.2524 ****	0.1547 ****		0.1227 ****
NO	0.1284 ****	−0.01743	−0.07064 ****	−0.07036 ****	−0.08121 ****	0.1939 ****	−0.2649 ****	−0.04592 *		0.05235 **
NO_2_	0.2098 ****	−0.04714 **	−0.1232 ****	−0.1166 ****	−0.1509 ****	0.09542 ****	−0.3754 ****	−0.0361 *		0.1572 ****
NOX	0.1922 ****	−0.0396 *	−0.1154 ****	−0.1105 ****	−0.1396 ****	0.1392 ****	−0.3629 ****	−0.03995 *		0.1340 ****
OX	−0.1190 ****	−0.1471 ****	0.04158 *	0.08215 ****	0.005769	−0.4305 ****	0.06489***	0.3078 ****		0.3264 ****
CH_4_	0.4844 ****	−0.1885 ****	−0.6326 ****	−0.6138 ****	−0.6537 ****	−0.3525 ****	−0.1902 ****	−0.03191		0.2223 ****
NMHC	0.1192 ****	−0.02196	−0.08328 ****	−0.08113 ****	−0.1018 ****	0.1358 ****	−0.2843 ****	−0.1009 ****		0.09666 ****
THC	0.3745 ****	−0.1304 ****	−0.4456 ****	−0.4326 ****	−0.4704 ****	−0.131 ****	−0.2986 ****	−0.08663 ****		0.1988 ****
SPM	−0.2336 ****	−0.03178	0.3668 ****	0.3747 ****	0.3503 ****	0.1566 ****	−0.2255 ****	0.1480 ****		0.07641 ****
PM_2.5_	0.03535	−0.1866 ****	0.05723 **	0.08374 ****	0.02575	−0.08667 ****	−0.2925 ****	0.2284 ****		0.2442 ****
					**Air pollutants**					
	SO_2_	NO	NO_2_	NOX	OX	CH_4_	NMHC	THC	SPM	PM_2.5_
**Meteorological data**										
Atmospheric pressure										
Precipitation										
Average temperature										
Highest temperature										
Lowest temperature										
Humidity										
Wind speed										
Hours of sunlight										
Temperature difference										
(day − previous day)										
**Air pollutants**										
SO_2_	1.0									
NO	0.4429 ****	1.0								
NO_2_	0.5645 ****	0.6765 ****	1.0							
NOX	0.5686 ****	0.8733 ****	0.9378 ****	1.0						
OX	−0.1249 ****	−0.5155 ****	−0.3193 ****	−0.4202 ****	1.0					
CH_4_	−0.1791 ****	0.1570 ****	0.3835 ****	0.3244 ****	0.1549 ****	1.0				
NMHC	0.4641 ****	0.7053 ****	0.7803 ****	0.8232 ****	−0.3153 ****	0.2655 ****	1.0			
THC	0.1839 ****	0.5491 ****	0.7364 ****	0.7276 ****	−0.1053 ****	0.7876 ****	0.8009 ****	1.0		
SPM	0.7039 ****	0.2567 ****	0.3614 ****	0.3540 ****	0.0431 *	−0.1848 ****	0.3421 ****	0.1027 ****	1.0	
PM_2.5_	0.6272 ****	0.3259 ****	0.5104 ****	0.4829 ****	0.1552 ****	0.2495 ****	0.4392 ****	0.4216 ****	0.8527 ****	1.0

* *p* < 0.05, ** *p* < 0.01, **** *p* < 0.0001. Abbreviations: SO_2_, sulfur dioxide; NO, nitric oxide; NO_2_, nitrogen dioxide; NOX, nitrogen oxides; CH_4_, methane; NMHC, non-methane hydrocarbons; THC, total hydrocarbons; SPM, suspended particulate matter; PM_2.5_, particulate matter (2.5 μm).

**Table 5:** Multivariate analysis of Poisson regression estimates measuring the association between the number of AE patients and environmental factors (2011–2019).

	Total (0–15 Years Old) (n = 7136)			0–5 Years Old (n = 4289)			6–15 Years Old (n = 2847)		
	Parameter	SE	*p*-Value	Parameter	SE	*p*-Value	Parameter	SE	*p*-Value
**Meteorological data**									
Atmospheric pressure (hPa)	0.0019	0.0061	0.75	0.0027	0.0080	0.73	0.0007	0.0096	0.94
Precipitation (mm)	0.0048	0.0036	0.19	0.0063	0.0046	0.17	0.0023	0.0058	0.69
Average temperature (°C)	0.0266	0.0090	0.00324 **	0.0315	0.0117	0.00711 **	0.0201	0.0143	0.16
Humidity (%)	0.0026	0.0039	0.51	0.0025	0.0050	0.63	0.0025	0.0062	0.69
Wind speed (m/s)	−0.029	0.026	0.26	−0.039	0.034	0.24	−0.013	0.040	0.73
Hours of sunlight (h)	0.0016	0.0094	0.87	−0.0019	0.0121	0.87	0.0064	0.0149	0.67
**Air pollutants**									
SO_2_ (ppm)	13.7	28.7	0.63	76.0	36.4	0.03682 *	−85.8	46.8	0.07
NO (ppm)	0.29	10.1	0.98	0.82	13.2	0.95	−0.8	16.0	0.96
NO_2_ (ppm)	−6.5	6.3	0.31	−14.9	8.1	0.065	6.8	10.3	0.51
OX (ppm)	6.6	4.9	0.17	7.4	6.3	0.24	5.5	7.8	0.48
CH_4_ (ppmC)	−0.3	1.0	0.81	0.68	1.3	0.61	−1.7	1.7	0.300
NMHC (ppmC)	−0.71	0.83	0.39	−1.20	1.08	0.27	−0.01	1.3	0.99
SPM (mg/m^3^)	0.009	0.0090	0.326	0.008	0.012	0.51	0.011	0.014	0.441
PM_2.5_ (µg/m^3^)	−0.010	0.012	0.401	−0.013	0.016	0.41	−0.007	0.019	0.73
**Infection**									
RSV prevalence	0.029	0.076	0.70	0.057	0.10	0.56	−0.009	0.12	0.94
Influenza prevalence	0.0045	0.0053	0.39	0.0058	0.0069	0.40	0.0031	0.0083	0.70
**Pollen (/m^3^)**	0.0008	0.0012	0.50	0.0015	0.0015	0.34	−0.0002	0.0020	0.93

* *p* < 0.05, ** *p* < 0.01. Abbreviations: AE, asthma exacerbation; SO_2_, sulfur dioxide; NO, nitric oxide; NO_2_, nitrogen dioxide; CH_4_, methane; NMHC, non-methane hydrocarbons; SPM, suspended particulate matter; PM_2.5_, particulate matter (2.5 μm); RSV, respiratory syncytial virus; ppmC, concentration unit of parts per million carbon.

## Data Availability

The datasets generated during and/or analyzed during the current study are available from the corresponding author on reasonable request.
